# Cost-Effective Use of Silver Dressings for the Treatment of Hard-to-Heal Chronic Venous Leg Ulcers

**DOI:** 10.1371/journal.pone.0100582

**Published:** 2014-06-19

**Authors:** Gregor B. E. Jemec, Jean Charles Kerihuel, Karen Ousey, Sanne Lise Lauemøller, David John Leaper

**Affiliations:** 1 Department of Dermatology, Health Sciences Faculty, Roskilde Hospital, University of Copenhagen, Copenhagen, Denmark; 2 Vertical, Paris, France; 3 School of Human and Health Sciences, University of Huddersfield, Huddersfield, United Kingdom; 4 Coloplast A/S, Humlebaek, Denmark; The University of Queensland, Australia

## Abstract

**Aim:**

To estimate the cost-effectiveness of silver dressings using a health economic model based on time-to-wound-healing in hard-to-heal chronic venous leg ulcers (VLUs).

**Background:**

Chronic venous ulceration affects 1–3% of the adult population and typically has a protracted course of healing, resulting in considerable costs to the healthcare system. The pathogenesis of VLUs includes excessive and prolonged inflammation which is often related to critical colonisation and early infection. The use of silver dressings to control this bioburden and improve wound healing rates remains controversial.

**Methods:**

A decision tree was constructed to evaluate the cost-effectiveness of treatment with silver compared with non-silver dressings for four weeks in a primary care setting. The outcomes: ‘Healed ulcer’, ‘Healing ulcer’ or ‘No improvement’ were developed, reflecting the relative reduction in ulcer area from baseline to four weeks of treatment. A data set from a recent meta-analysis, based on four RCTs, was applied to the model.

**Results:**

Treatment with silver dressings for an initial four weeks was found to give a total cost saving (£141.57) compared with treatment with non-silver dressings. In addition, patients treated with silver dressings had a faster wound closure compared with those who had been treated with non-silver dressings.

**Conclusion:**

The use of silver dressings improves healing time and can lead to overall cost savings. These results can be used to guide healthcare decision makers in evaluating the economic aspects of treatment with silver dressings in hard-to-heal chronic VLUs.

## Introduction

Chronic venous, lower limb ulceration affects 1–3% of the adult population worldwide [1] and some patients suffer a repeated cycle of ulceration, healing, and recurrence. These ulcers will take months to heal despite appropriate treatment, including efficient venous compression bandage systems [1]–[3] and have 12-month recurrence rates between 18% and 28% [4], [5]. Ulcer size and ulcer duration are clearly identified risk factors for a poor healing prognosis [6]. Venous leg ulcers (VLUs) are also frequently painful, malodourous, often with moderate to high exudate, and have a significant negative impact on patients’ quality of life [7], [8]. Treatment is associated with considerable costs to healthcare systems [4]. The underlying pathogenesis of these hard-to-heal VLUs is complicated by excessive and prolonged inflammation which is often related to critical colonisation and early localised infection [9], [10]. A heavy bioburden of colonizing microorganisms in the wound may be one of the most important barriers to wound closure [11]. Ionized silver (Ag^+^) has both anti-inflammatory and antimicrobial properties with a broad spectrum of action [12]–[14]. The use of silver-releasing dressings to control bioburden and improve VLU healing rates has been the subject of considerable debate with diverse conclusions [15]–[20]. The VULCAN trial published in 2009 [21] showed no difference in VLU healing rates over 12 weeks of observation, when comparing treatment with silver dressings or non-silver containing absorptive dressings. Following this publication the use of silver was not recommended because of the associated higher cost [21], but no measurement of VLU microbial colonisation or the antimicrobial effect of silver dressings was made in the trial. However, a recent meta-analysis showed a statistically significant treatment effect, responder rate, and healing rate in favour of a silver dressing when treating critically colonised VLU for four weeks compared with non-silver dressings [22]. The latter results are in accordance with the guidance of an international consensus group which recommend that silver dressings should be used when a VLU becomes troublesome (hard-to-heal) and critical colonisation is suspected or has progressed to localised infection [23]. All silver dressings are only indicated for their effect to reduce critical colonisation; none are recommended for healing of VLUs when used without other supportive treatments, and particularly when there are no clinical signs of progressive colonisation.

The aim of the current study was to estimate the cost-effectiveness of silver dressings when used according to recommendations [24]. This was undertaken using a health economic model based on the time-to-wound-healing in hard-to-heal chronic VLUs which showed signs of critical colonisation or early localised infection. Evidence of a cost-effective benefit could be helpful for healthcare decision makers in evaluating the economic aspects of treatment with silver-releasing dressings.

## Methods

### Decision Analytic Framework

**Figure 1:**
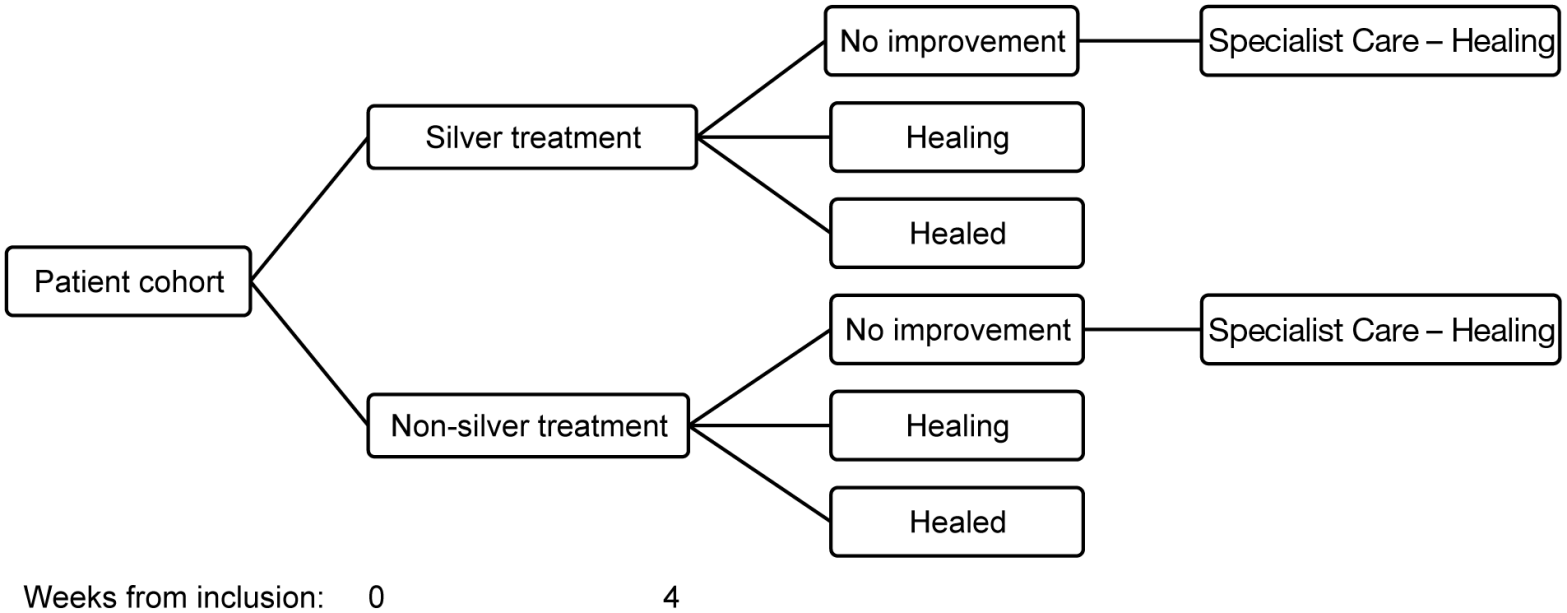
Framework for health economic model. The patient cohort consisted of 659 patients with hard-to-heal VLUs.

In order to establish a health economic model a decision analytic framework (decision tree) was constructed (Figure 1). The decision tree explored a decision to use silver dressings (‘Silver treatment’) or non-silver dressings (‘Non-silver treatment’) for four weeks in a primary care setting. If ulcers did not improve during this four week period the patients were assumed to have been referred to specialist care (Figure 1). The model also reflected ulcer management in primary care where it was assumed that a silver treatment would be the first type of dressing used for patients with hard-to-heal VLUs.

During the initial four week period each patient was deemed to have one of three possible outcomes; complete healing (‘healed ulcer’), the ulcer may have decreased in size (‘healing ulcer’), be unchanged or enlarged (‘no improvement’). The proportion of patients in each outcome category was estimated: the outcome of ‘healed ulcer’ at four weeks was based on the definition of wound healing [25], [26] used in the four clinical trials included in a published meta-analysis [22]. ‘No improvement’ was defined as those ulcers with no change or an expanded ulcer area from baseline to week four of surveillance. Remaining patients had a decrease in ulcer area but did not have a healed VLU at four weeks; this group was categorized as having a ‘healing ulcer’ in the context of the cost calculation. The progression of ulcer healing after the four week initial treatment could not be estimated directly from the clinical data set. Hence, a number of simplifying assumptions were made:

1. Patients who responded to the four weeks treatment were assumed to have continued treatment with a non-silver treatment until their ulcer was healed. Time to wound healing was estimated by linear extrapolation of the ulcer areas at baseline and at four weeks for each patient in the data set.
2. Patients who did not respond after four weeks treatment were assumed to have been referred to a wound specialist for wound assessment and development of a treatment plan. The healing time for these ulcers was assumed to be the same whether the patient was started on the silver treatment or the non-silver treatment, and set equal to the estimated healing time in patients with improved ulcers at four weeks in the silver treatment.

### Cost of Wound Management

The cost of wound management was assessed from a United Kingdom (UK) National Health Care perspective for up to one year after the VLU treatment had started. To estimate the cost of wound management a number of simplifying assumptions were made:

1. Ulcer management was assumed to be performed in a primary care setting.
2. Nurse salary rates were based on that of a community nurse.
3. Dressing costs included the use of an absorbent dressing. Costs of absorbent dressings were based on 10×10 cm sizes, which are the most commonly used sizes.
4. A hard-to-heal ulcer was assumed to be changed four times a week and a normally healing ulcer only twice a week.
5. Patients who did not improve at four weeks were referred to a wound specialist for further investigation. This was modelled as a “one off” cost, assuming that the patient would be reviewed by a wound specialist nurse (1 hour appointment including urine test, Ankle Brachial Pressure Index (ABPI) measurement, assessment of treatment plan, communication with primary care nurses, and follow-up).
6. A band 6 nurse salary rate was used for the estimation of the cost of this visit.
7. 10% of patients attending the wound clinic were assumed to have been referred for further investigation; in this assumption to include duplex Doppler scanning.
8. After referral to a wound specialist, patients were treated in primary care where their VLU was considered to be hard-to-heal for an additional two weeks (as opposed to the four weeks treatment with silver). After that the patient would be considered as having the same healing time as patients treated with silver.

Table 1 provides an overview of unit cost applied in the cost estimation.

**Table 1:** Unit cost applied in the analyses (£).

Item	Value (£)	Source
Biatain dressing (10×10)	2.35	Drug tariff price. September 2013
Biatain Ag dressing (10×10)	7.97	Drug tariff price. September 2013
Primary care visit (incl. transportation, 1 h)	31.00	Cost of Clinical Support Worker Nursing per hour taken from PSSRU 2012 (p.188)
Secondary care		
Initial assessment	103.47	NHS reference costs 2012 (outpatient currency code 107; first visit)
Follow-up visit	84.04	NHS reference costs 2012 (outpatient currency code 107; follow-up)
ABPI assessment	29.95	NHS reference costs 2012 (outpatient currency code 182; first visit)
Duplex scan	55.01	Weighted mean from NHS reference costs 2012 (Total HRGs; RA23Z–RA27Z)

### Data Sources

To estimate the cost of wound management data was sourced from the clinical trial data in a published meta-analysis [22]. The data set was based on four RCTs conducted on 685 patients where the same active silver dressing (Biatain Ag, Coloplast) was compared with non-silver dressings with respect to relative reduction in ulcer area at four weeks. The meta-analysis showed a statistically significant treatment effect (p = 0.0001), responder rate (p = 0.001) (defined as the proportion of patients with a relative ulcer area reduction ≥40% at 4 weeks), and healing rate (p = 0.002) in favour of the silver dressing. All patients had venous or mixed aetiology leg ulcers that exhibited delayed healing (defined as clinical signs of infection (pain, odour, increased exudate) and/or less than a 20% ulcer size reduction over four weeks). These ulcers were defined as hard-to-heal VLUs.

### Sensitivity Analysis (Tornado Diagram)

**Figure 2:**
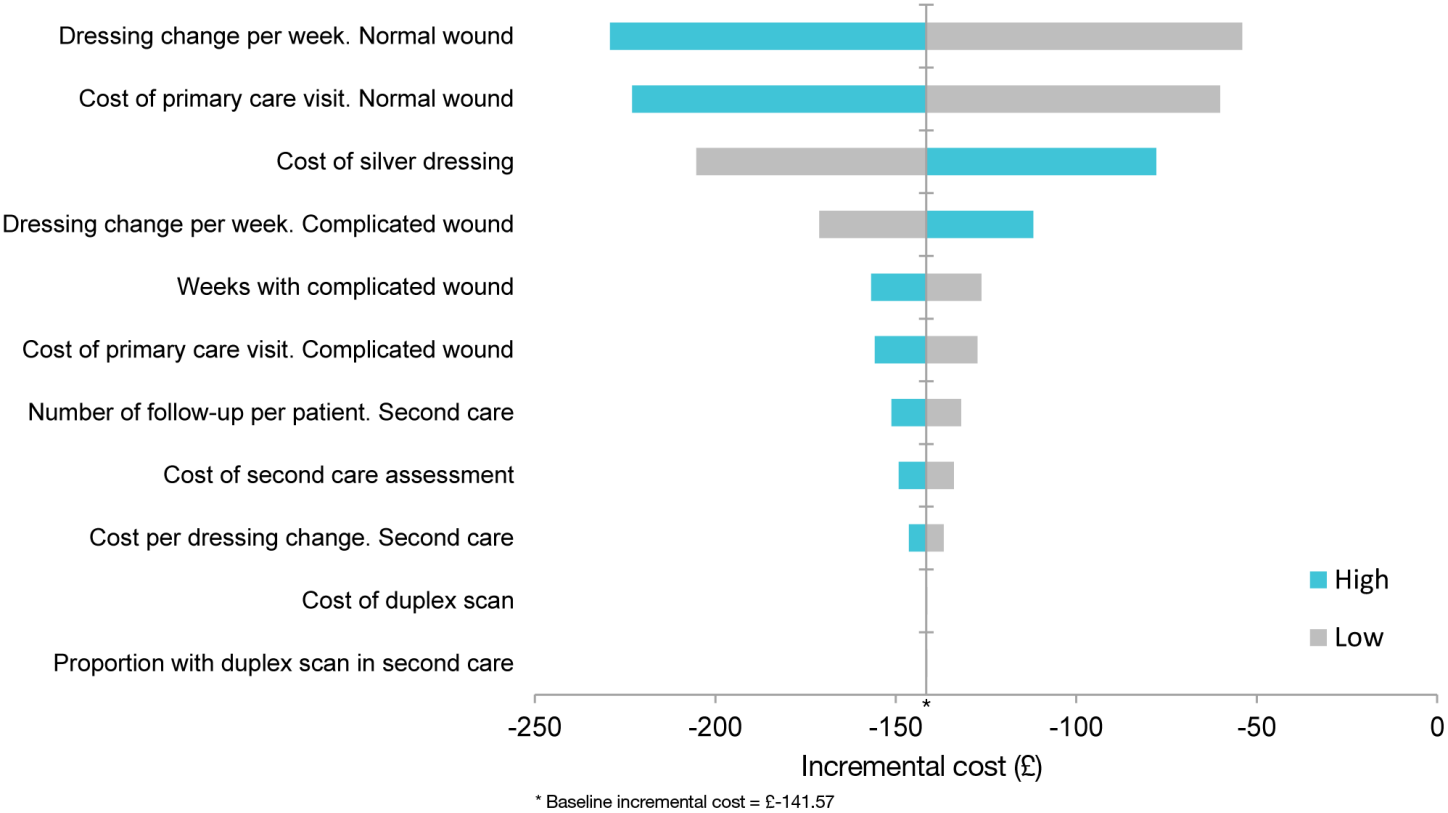
Sensitivity analysis. Change in incremental cost (£) per patient (silver treatment versus non-silver treatment) when changing key assumptions ±50%. The figure includes the assumptions that ‘Dressing change per week. Normal wound’; ‘Cost of primary care visit. Normal wound’; ‘Cost of silver dressing’; have the highest impact on the incremental cost per patient. For example if the ‘cost of silver dressing’ was higher (turquoise bar) the incremental cost per patient would be reduced, nevertheless, even if the price of silver dressing is 50% more expensive the incremental cost would remain below zero (i.e. be cost-saving).

The estimation of treatment costs of silver dressings was based on typical treatment patterns and did not rely on observed health care utilisation. In order to assess how robust the silver treatment cost estimates were with respect to the key assumptions and unit cost established by this model, a univariate sensitivity analysis was conducted. Each of the unit costs listed in Table 1 was varied by ±50%. Dressing change per week was tested in the range 1–3 changes per week (baseline 2 per week) for a normally healing wound and in the range 3–5 changes per week (baseline 4 per week). The Tornado diagram (Figure 2) illustrates which model parameter has the largest impact on estimated differences in treatment cost.

### Statistical Methods

The statistical uncertainty in the estimated cost difference between the two treatment arms was estimated from the pooled data set. Using the health economic framework an estimate of wound management cost was assigned to each patient and 95% confidence intervals (CI) were calculated for the difference in cost between the two treatment arms. No statistical test was performed.

### Ethic Statement

The study design is based on treatments algorithms, cost estimations, and analysis of already published data from a meta-analysis, and therefore no ethics committee approval was relevant or sought.

## Results

### Clinical Outcomes

A higher proportion of ulcers treated with silver dressings healed during the four week period compared with wounds treated with non-silver dressings (7.6% compared with 3.4%). The proportion of healing ulcers was also higher in the group treated with silver compared with non-silver dressings (79.4% compared with 72.1%, respectively). A lower proportion of patients treated with silver dressings had no improvement in ulcer area during the four weeks compared with patients treated with non-silver dressings (13.0% compared with 24.5%, respectively, Table 2).

**Table 2:** Patient outcome after four weeks silver dressing compared with non-silver wound management in pooled data set from four clinical trials.

	N	Response classification (%)			Additional weeks to healed ulcer*		
		Healed ulcer†	Healing ulcer†	No improvement‡	N	Average	Median
Group							
Silver	369	7.6	79.4	13.0	293	10.1	4.9
Non-silver	290	3.4	72.1	24.5	209	12.8	6.4

*Applies to 'Healing ulcer' only. Number of weeks after week 4. Estimates truncated at 1 year.

† Data from meta-analysis [22].

‡ Unpublished data.

The estimated healing time for the VLUs treated with silver dressings was shorter than the healing time for the non-silver treatment group with an average of 10.1 weeks compared with 12.8 weeks, respectively, Table 2.

### Economic Results

The economic evaluation of the four week silver treatment in primary care compared with non-silver treatment is shown in Table 3.

**Table 3:** Comparison of cost of wound management (£) using a four week silver treatment compared with non-silver treatment in primary care.

	Patients (%)	Resources (weeks)	Unit cost	Cost per patient (£)
**Silver treatment**				
Initial 4 weeks (primary care)	100	4†	155.88 £ per week	623.52
Additional treatment in primary care				
Healed ulcer	7.6	-	0.00 £ per patient	-
Healing ulcers*	79.4	10.1‡	66.70 £ per week	534.44
No improvement (referred to specialist)	13.0			
Initial assessment/follow-up**			222.96 £ per patient	29.00
Wound management. Complicated wound		2†	133.40 £ per week	34.71
Wound management. Healing wound***		12.1‡	66.70 £ per week	104.91
Total cost per patient				1,326.57
Average estimated time to healed wound		13.8		
**Non-silver treatment**				
Initial 4 weeks (primary care)	100	4†	133.40 £ per week	533.60
Additional treatment in primary care				
Healed ulcer	3.4	-	0.00 £ per patient	-
Healing ulcers*	72.1	12.8‡	66.70 £ per week	617.19
No improvement (referred to specialist)	24.5			
Initial assessment/follow-up**			222.96 £ per patient	54.59
Wound management. Complicated wound		2†	133.40 £ per week	65.32
Wound management. Healing wound***		12.1‡	66.70 £ per week	197.44
Total cost per patient				1,468.14
Average estimated time to healed wound		16.7		
**Incremental cost**				−141.57

*Based on linear extrapolation of wound closure during first 4 weeks observed in the meta-analysis [22].

**Unit cost of initial assessment/follow up (From Table 1: £103.47 initial assessment + £84.04 follow-up visit + £29.95 ABPI assessment + £5.50 Duplex scan (10% of patients assumed to be referred to duplex scan)).

***Total healing time was assumed equal to average time to healing in patients with non-expanding wound estimated in the meta-analysis (minimum of healing time estimated for silver treatment respectively non-silver treatment arm). The split between weeks with complicated wound and normally healing wound was equal in both silver treatment and non-silver treatment arms.

† High frequency dressing change (4 times/week).

‡ Low frequency dressing change (2 times/week).

The initial four weeks treatment in primary care was estimated to be more expensive for the group treated with silver (£623.52) compared with non-silver treatment (£533.60). Nevertheless, a higher proportion of patients treated with silver had ulcers with complete healing or healing ulcers, and therefore the estimated average time-to-healed wound was lower (13.8 weeks) compared with non-silver treatment (16.7 weeks). Hence the average total treatment cost per patient was lower for silver treatment (£1,326.57) compared with non-silver treatment (£1,468.14) with a cost saving of £141.57 (Table 3).

The use of a four week silver treatment was considered to be cost saving because of a shorter time to healing, and fewer patients requiring referral to specialist care.

In order to assess how robust the silver dressing cost estimate was, with respect to the key assumptions and unit cost established by this model, a univariate sensitivity analysis was conducted (Figure 2). Within the tested range for the modelling parameters the incremental cost of the silver treatment was below zero (i.e. cost-saving) (Figure 2). Thus the health economic model seemed to be robust to modelling assumptions.

## Discussion

Venous ulceration with clinical signs of critical colonisation/local infection, which forms the focus of this study, is of a chronic nature and therefore can be classified as hard-to-heal. The time and costs taken by the health care professionals to diagnose, review and prescribe, when added to the nurse time for wound management, dressing changes and application of compression bandaging, is considerable and outweigh the cost of individual dressings [27], [28]. This health economic analysis was developed in order to estimate the cost-effectiveness of a more expensive silver dressing compared with a lower-priced non-silver absorbent dressing, using the best available clinical data [22]. The model divides the outcomes into three categories (healed, healing, and no improvement) and the costs of VLU management reflect a UK National Health Care perspective. Treatment with silver dressings for an initial period of four weeks, using the economic model, was associated with a cost saving (£141.57, CI 95% from −275.97 to −7.24) compared with the group of patients treated with non-silver dressings. This was related to a higher healing rate and shorter time for wound closure (13.8 weeks compared with 16.7 weeks, respectively). A univariate sensitivity analysis on key assumptions supported these results. Thus, this health economic analysis seeks to quantify the cost-implications arising from using a silver dressing by applying a modelling approach.

Furthermore, using a modelling approach may be better than a prospective study such as the VULCAN trial [21]. The VULCAN trial has been criticised for being both too broad and using an irrelevant treatment protocol (heterogeneous use of silver dressings with varying contents and different release rates of silver ions, inappropriate long-term use of silver dressings, use of silver dressings to heal VLUs rather than their use to control bioburden; and having no clinical or microbiological assessment of bioburden made) [29]. Hence, any treatment effect found in the VULCAN trial does not reflect recommended uses of silver dressings by their manufacturers [30]. Measuring resource utilisation can be complicated in prospective studies (for example the VULCAN trial indirectly estimated the cost of dressing use). We have applied a modelled-cost outcome based on the best available clinical data consisting of 659 patients with hard-to-heal VLUs with clinical signs of infection. The patients were treated with a silver dressing or a non-silver dressing for four weeks [22] and thereby represent the best option for calculating the cost implication of using silver dressings appropriately.

Compared with other modelling-based approaches which have addressed the cost-effectiveness of silver dressings [31] this analysis included more robust, clinical (pooled) data [22]. In addition, we have presented the finding from a meta-analysis that silver dressings do not only save cost because of a shorter time to healing (as shown in [31]) but also by reducing the number of patients with no wound closure or enlarging ulcers (unpublished data); these patients need special attention in clinical practice and are likely to be costly to manage.

Although the results presented here are not based on prospectively-collected, resource utilisation data, the findings indicate that the use of the silver-challenge in hard-to-heal leg ulcers is cost-saving compared with the use of non-silver dressings.

Limitations of the study: in this health economic model, it was assumed that all ulcers would eventually heal (based on linear extrapolation of healing time). However, in reality some ulcers may become re-infected and progress to more serious infections, or other complications which could have arisen, and require additional wound management and care. The clinical data applied this model was a 4 weeks period of silver treatment; a shorter challenge period of silver as suggested by the consensus group [23] or a longer follow up period was not allowed to be entered into the model.

## Conclusion

Based on a health economic model, where clinical data was sourced from a recently published meta-analysis, it has been shown that when patients with hard-to-heal VLUs are allocated to an initial four weeks treatment using silver dressings there can be associated cost savings (£141.57) compared with patients who are treated with non-silver dressings. In addition, patients treated with silver dressings had wound closure approximately 3 weeks before those patients treated with non-silver dressings. Thus, use of silver dressings improves healing time in hard-to-heal VLUs and can lead to overall cost-savings. These results can be used to guide healthcare decision makers in evaluating the economic aspects of treatment with silver dressings in hard-to-heal VLUs.
